# Optic nerve sheath diameter measurement for the paediatric patient with an acute deterioration in consciousness

**DOI:** 10.1186/s13089-023-00341-6

**Published:** 2023-12-04

**Authors:** Ahmed Ali, David J. McCreary

**Affiliations:** Paediatric Emergency Department, Sunderland Royal Hospital, South Tyneside and Sunderland NHS Foundation Trust, Sunderland, UK

**Keywords:** Paediatrics, POCUS, Optic nerve sheath diameter, Intracranial pressure, Subdural empyema

## Abstract

Ocular Point of Care Ultrasound (PoCUS) is emerging as a valuable utility within emergency medicine. Optic nerve sheath diameter (ONSD) has been demonstrated to correlate closely with intracranial pressure (ICP) and an elevated measurement can detect raised ICP readily, where fundoscopy may not, owing to both technical challenges and insufficient clinical skills. A previously fit and well 10-year-old girl presented to the paediatric emergency department with worsening headache, fever and lethargy. On examination, her left pupil was large, and not reactive to light. Initially, her GCS was 15 but suddenly dropped to 8/15. Her blood tests showed raised inflammatory markers. A CT head was reported as possible pansinusitis and MRI of her brain was initially reported as showing evidence of meningeal irritation only. Due to her drop in GCS PoCUS of optic nerve sheath was conducted which showed evidence of increased ICP with increased optic nerve sheath diameter of 6.8mm. This led to a reassessment of the MRI imaging by the neurosurgical team who felt there was evidence of subdural empyema. The patient was transferred to the tertiary neurosurgical centre, where an emergency evacuation of subdural empyema was carried out. Staphylococcus aureus and Streptococcus pyogenes were grown from pus samples. Early detection of raised ICP is of paramount importance in terms of being able to instigate neuroprotective measures and prevent adverse neurological outcomes. PoCUS is a readily available, non-irradiating, easily repeatable, well-tolerated and readily teachable ultrasound modality and a useful tool which should be employed in paediatric and adult emergency departments.

## Case

A previously fit and well 10-year-old girl initially presented to the paediatric emergency department (PED) complaining of sore throat and painful swallowing without fever. She was discharged with a diagnosis of upper respiratory tract infection. Five days later she developed a high-grade fever of up to 38.8C associated with lethargy and left-sided headache. In the 24 h before representing to the PED, her headache was severe, frontal, constant and associated with dizziness. The only other relevant history was being hit in the left side of the face by a basketball 2 days prior which did not result in loss of consciousness. On examination, she was afebrile with normal blood pressure for her age and height. She was alert (GCS 15/15); however, she looked unwell. She was noted to have a subtle left eyelid ptosis and her left pupil was large and not reactive to light. There was no redness or chemosis of the eye. When assessing eye movements she demonstrated limited infraduction and supraduction of the left eye. The right eye exam was normal along with the remainder of her cranial nerves. Neurological examination of all four limbs was also normal.

Initial blood tests revealed a significantly elevated white cell count at 43.9 × 10′ 9/L, with a neutrophil of 42.6 × 10′ 9/L, with normal haemoglobin and normal platelet count. CRP was elevated at 257.6 mg/L. Her sodium was low at 131 mmol/L with normal potassium, urea and creatinine.

**Fig. 1:**
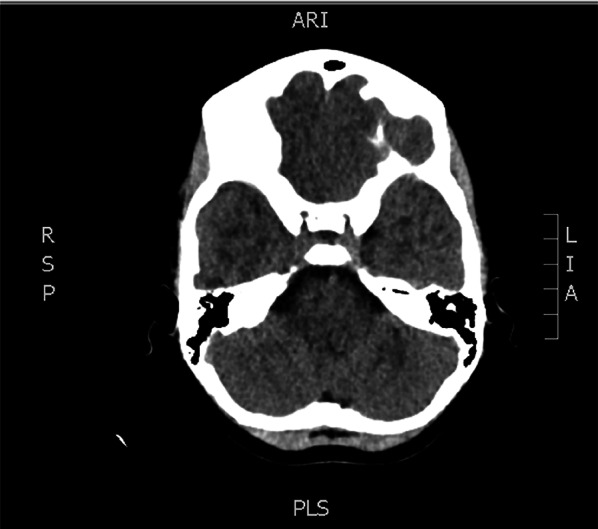
Patients CT scan showing normal cerebral parenchyma with no evidence of haemorrhage, space-occupying lesion, infarct, or intra or extra-axial fluid collections

**Fig. 2:**
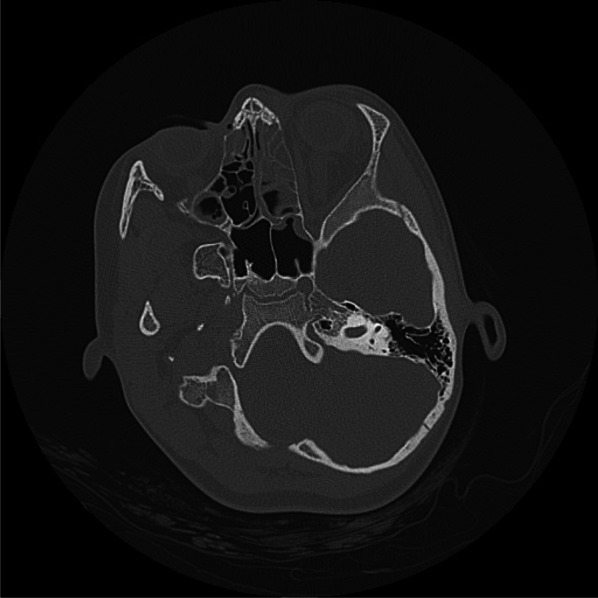
Patients CT scan showing opacification of the left frontal, ethmoidal and both maxillary sinus

She underwent a head CT (Figs. 1, 2) which demonstrated apparent normal cerebral parenchyma with no evidence of haemorrhage, space-occupying lesion, infarct, or intra or extra-axial fluid collections. The only positive findings were opacification of the left frontal, ethmoidal and both maxillary sinus with a likely inflammatory origin. The reporting radiologist recommended an MRI brain with DWI sequences if clinical concerns persist.

**Fig. 3:**
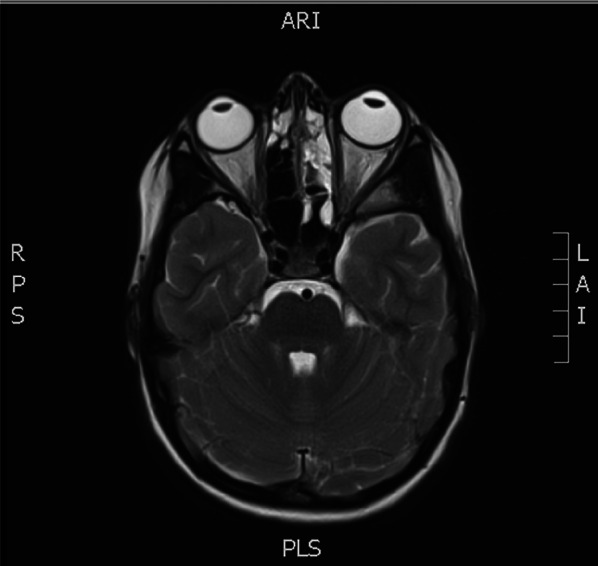
Patients MRI scan demonstrating paranasal sinusitis with near complete opacification of the left frontal, ethmoidal and maxillary sinuses and further mucosal thickening in the left posterior ethmoidal and sphenoidal sinus

Due to the severity of this patient's symptoms and in view of her blood results an MRI of her brain and orbits was conducted (Fig. 3). This demonstrated evidence of paranasal sinusitis with near complete opacification of the left frontal, ethmoidal and maxillary sinuses and further mucosal thickening in the left posterior ethmoidal and sphenoidal sinuses. No intraorbital fluid collection was identified. The remainder of the report was as follows: there is a degree of prominence of the CSF sheath around the optic nerves and also a subtle posterior bulge at the optic nerve head. On the FLAIR images, there is subtle sulcal hyperintensity seen which is more prominent on the left side involving the frontal, parietal and occipital and to a lesser extent the temporal lobes regions. There is also subtle hyperintensity along the surface of the left cerebral hemispheres on the FLAIR images which corresponds to minor diffusion restriction seen on the diffusion-weighted images. In view of the clinical history, an inflammatory meningeal component is suspected and as needed a post-contrast study to confirm or refute these findings can be performed. There is no intracranial extra-axial fluid collection shown.

**Fig. 4:**
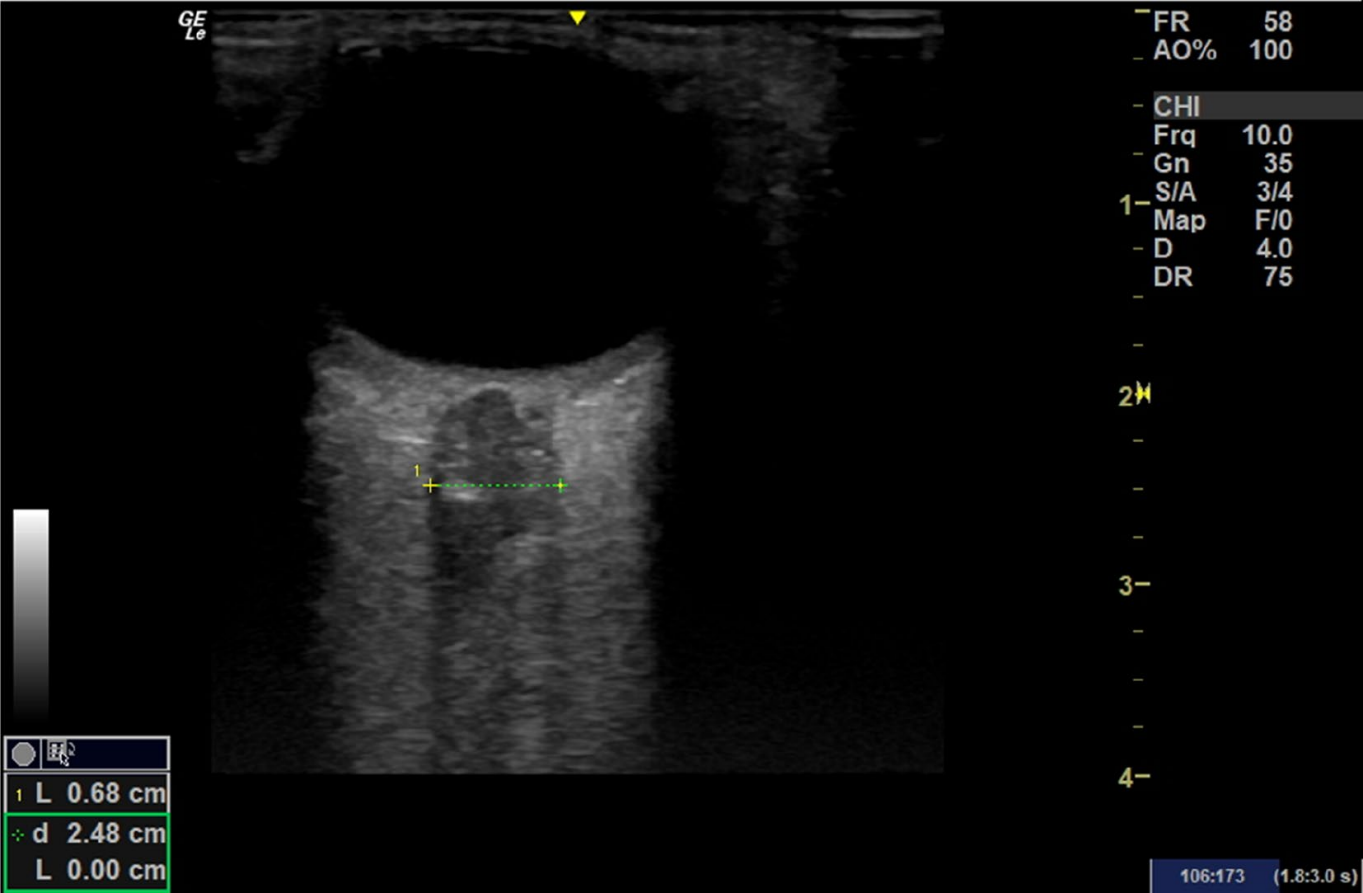
Patients ocular POCUS showing an increased optic nerve sheath diameter (ONSD) of 6.8 mm with no current evidence of papilloedema

The patient suffered an acute drop in GCS on return from her MRI scan. The supervising consultant trained in Point of Care Ultrasound, reviewed her for the first time and performed an ocular POCUS (Fig. 4). Her optic nerve sheath diameter was 6.8 mm (expected normal 5–5.5 mm) suggesting raised intracranial pressure. There was no evidence of papilloedema. The POCUS findings prompted a review of the MRI imaging by the tertiary neurosurgical team based on a different hospital who were significantly concerned about intracranial extension and formation of subdural empyema. They recommended a time-critical transfer to the neurosurgical centre.

Having already received IV cefotaxime and acyclovir, after her POCUS she was given a dose of 2.7% hypertonic saline 3 ml/kg over 30 min. After an urgent transfer to the tertiary centre, she underwent left fronto-temporo-parietal decompressive craniectomy and evacuation of subdural empyema which occurred without parental consent owing to the time-critical nature of her condition. Thankfully she was extubated the following day without any evidence of neurological impairment. A repeat MRI 4 days later showed no evidence of residual empyema. Microbiology results from pus samples taken at the time grew Staphylococcus Aureus and Streptococcus pyogenes.

### Subdural empyema in children

Subdural Empyema (SDE) is a intracranial purulent collection between the dura and arachnoid mater. The mortality rate of patients with SDE is around 4%, while the morbidity for survivors is even higher with residual neurologic deficits reaching up to 50%, hemiparesis 15–35% and persistent seizures 12–37.5% [1]. Patients may present with the typical symptoms of sinusitis-like fever, headache and purulent rhinorrhoea along with photophobia and painful paresthesia in the area covered by the trigeminal nerve. Overall, patients with SDE due to frontal sinusitis have more subtle signs and symptoms compared to infection of other sinuses [2]. Magnetic resonance imaging (MRI) has a sensitivity of 93% and is considered the best imaging mode for SDE, because it is able to demonstrate the collections and signs of meningeal infection [3]. Conservative treatment is recommended for patients with non-focal neurological deficits, no changes in mental status and if the response to antibiotics is adequate [4]; however, craniotomy may be required for evacuation [5].

### Use of POCUS for raised intracranial pressure

Anatomically the optic nerve sheath is contiguous with the subarachnoid space meaning that an increase in ICP results in a corresponding increase in the optic nerve sheath diameter [6].

**Fig. 5:**
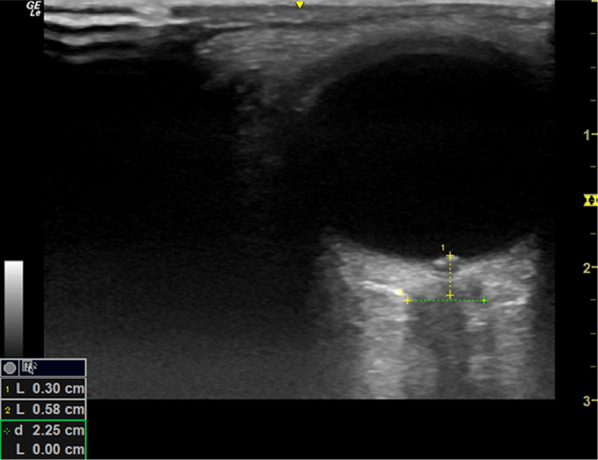
Ocular POCUS showing the site of measurements for optic nerve sheath diameter (ONSD). This image demonstrates and increased ONSD and papilloedema

To perform ocular POCUS select the high-frequency linear probe. A Tegaderm or other suitable film covering should be placed over the patient’s closed eye. A generous amount of ultrasound gel is required to allow for optimal transmission of the ultrasound waves and to ensure excessive pressure is not transmitted to the globe. With this in mind ocular POCUS is contraindicated in the setting of globe injury or where further direct pressure may lead to worsening of existing ocular damage. The examination is well-tolerated by patients including young children provided a clear explanation of what is involved is provided. Anchoring your fingers or heel of the hand gently on the patient’s forehead or cheeks helps improve image acquisition and maintains it steadily in view. A transverse probe orientation is required to visualise the optic nerve. The aim is to visualise the anterior structures of the eye and then rock the probe laterally and slightly inferiorly until the optic nerve is in view. A measurement is taken 3mm posterior to the interface of the retina and the optic nerve as show in Fig. 5 [6].

Sonography of the ONSD has been demonstrated to be both a sensitive test for ruling out raised ICP in low-risk patient groups as well as a specific test for ruling in raised ICP in higher risk groups such as those with a reduced or fluctuating GCS such as those with ventricular shunt requiring evaluation, perioperative neurosurgical patients or those suffering traumatic brain injury [7].

**Fig. 6:**
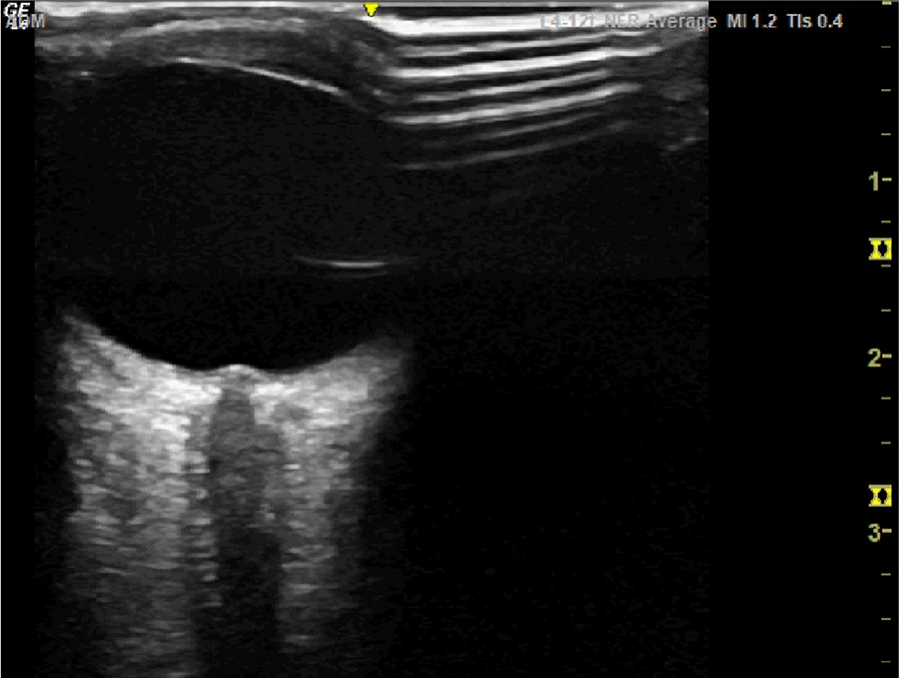
Ocular POCUS demonstrating papilloedema with anterior protrusion of the optic nerve head > 1 mm

Ocular POCUS performs very well in terms of diagnostic accuracy for detection of raised ICP compared with CT with a sensitivity of 95.6% (95% CI 87.7–98.5%) and specificity of 92.3% in one recent systematic review [8]. Remarkably the ONSD has been demonstrated to respond to increases in intracranial pressure in real time and thus is considered to be the best non-invasive modality for dynamic estimation of ICP [9]. To ensure values are as accurate as possible it has been suggested that multiple measurements are taken and averaged out [10]. Papilloedema can be readily appreciated even by the less experienced user and is typically defined as an elevation of the optic disc by > 1 mm, shown as an anterior protrusion like in Fig. 6 [11].

Early detection of raised ICP is of paramount importance in terms of being able to instigate neuroprotective measures and prevent adverse neurological outcomes. POCUS is readily available, non-irradiating and easily repeatable which means that more timely interventions can occur for the evolving patient suspected of having raised ICP. It would, therefore, be most useful in centres, where timely, out-of-hours CT reporting is challenging or during patient transport en route to intensive care settings [12].

Fundoscopy remains a challenging skill, particularly in paediatric patients and without dilatation of the pupil, meaning that important findings may be easily missed [13]. One reference intimates that “many medical students never view an abnormal fundus” via fundoscopy [14]. Ocular POCUS on the other hand is a quick, well-tolerated and readily teachable ultrasound modality and a useful tool which should be employed in paediatric and adult emergency departments.

## Data Availability

Not applicable.
